# Curcumin Inhibits CD4^+^ T Cell Activation, but Augments CD69 Expression and TGF-β1-Mediated Generation of Regulatory T Cells at Late Phase

**DOI:** 10.1371/journal.pone.0062300

**Published:** 2013-04-26

**Authors:** Girak Kim, Mi Seon Jang, Young Min Son, Min Ji Seo, Sang Yun Ji, Seung Hyun Han, In Duk Jung, Yeong-Min Park, Hyun Jung Jung, Cheol-Heui Yun

**Affiliations:** 1 Department of Agricultural Biotechnology and Research Institute for Agriculture and Life Sciences, Seoul National University, Seoul, Republic of Korea; 2 Department of Oral Microbiology & Immunology, BK21 Program, and Dental Research Institute School of Dentistry, Seoul National University, Seoul, Republic of Korea; 3 Konkuk University College of Medicine, Seoul, Republic of Korea; 4 National Institute of Animal Science, Suwon, Gyeonggi-do, Republic of Korea; 5 Center for Food and Bioconvergence, Seoul National University, Seoul, Republic of Korea; 6 World Class University Biomodulation Major, Department of Agricultural Biotechnology, Seoul National University, Seoul, Republic of Korea; Charité-University Medicine Berlin, Germany

## Abstract

**Background:**

Curcumin is a promising candidate for a natural medicinal agent to treat chronic inflammatory diseases. Although CD4^+^ T cells have been implicated in the pathogenesis of chronic inflammation, whether curcumin directly regulates CD4^+^ T cells has not been definitively established. Here, we showed curcumin-mediated regulation of CD2/CD3/CD28-initiated CD4^+^ T cell activation *in vitro*.

**Methodology/Principal Findings:**

Primary human CD4^+^ T cells were stimulated with anti-CD2/CD3/CD28 antibody-coated beads as an *in vitro* surrogate system for antigen presenting cell-T cell interaction and treated with curcumin. We found that curcumin suppresses CD2/CD3/CD28-initiated CD4^+^ T cell activation by inhibiting cell proliferation, differentiation and cytokine production. On the other hand, curcumin attenuated the spontaneous decline of CD69 expression and indirectly increased expression of CCR7, L-selectin and Transforming growth factor-β1 (TGF-β1) at the late phase of CD2/CD3/CD28-initiated T cell activation. Curcumin-mediated up-regulation of CD69 at late phase was associated with ERK_1/2_ signaling. Furthermore, TGF-β1 was involved in curcumin-mediated regulation of T cell activation and late-phase generation of regulatory T cells.

**Conclusions/Significance:**

Curcumin not merely blocks, but regulates CD2/CD3/CD28-initiated CD4^+^ T cell activation by augmenting CD69, CCR7, L-selectin and TGF-β1 expression followed by regulatory T cell generation. These results suggest that curcumin could directly reduce T cell-dependent inflammatory stress by modulating CD4^+^ T cell activation at multiple levels.

## Introduction

Curcumin has been reported to exhibit a variety of immunoregulatory functions [1]–[4], including induction of maturation arrest or a tolerogenic state in dendritic cells (DCs), and subsequently enhancing regulatory T cell differentiation [5], [6]. Moreover, curcumin can directly induce T cell apoptosis at high dose as well as inhibit T cell activation through blockade of the IL-2 signaling pathway and/or inhibition of mitogen-initiated activation of NF-κB and AP-1 [7]–[11]. Curcumin also regulates T cell response to IL-12 by inhibition of Th1 differentiation through blockade of JAK-STAT signaling activation [12], [13]. However, some reports showed that curcumin increases T lymphocyte proliferation and inhibits T cell apoptosis induced by dexamethasone or UV irradiation [14]–[16]. Thus, precise action mechanism of the immunological influence of curcumin on CD4^+^ T cells remains to be determined.

Curcumin attenuates the severity of a variety of chronic inflammatory diseases, including different forms of cancer, allergic reactions, asthma, inflammatory bowel disease, rheumatoid arthritis and Alzheimer’s disease [17], [18]. The therapeutic efficacy of curcumin has been mainly associated with down-regulation of the expression of proinflammatory cytokines such as TNF-α/β, IL-1, IL-6 and IL-8, and cyclooxygenase-2 [19], [20]. It is also likely that curcumin’s therapeutic efficacy would also have in relation to the regulation of CD4^+^ T cell activity, considering CD4^+^ T cell-driven inflammatory stress in the pathogenesis of chronic inflammation [21].

Recent studies suggest that CD69 negatively regulate the development of chronic inflammatory diseases [22]–[24]. While CD69 signaling induces TGF-β protein synthesis in NK cells, macrophages and CD3^+^ T lymphocytes [22], [25], it also inhibits sphingosine 1-phosphate receptor-1, which is required for lymphocyte egress from lymph nodes, effectively suppressing leukocyte infiltration in response to localized inflammation [26], [27] Interestingly, CD69 appears to be persistently expressed on the infiltrating CD4^+^ T cells during chronic inflammatory diseases [28], [29], suggesting that CD69 may also regulate chronic inflammatory conditions through concomitant TGF-β biosynthesis and inhibition of leukocyte egress [22]–[24], [27]. Furthermore, it was recently reported that CD69 activation of JAK3-STAT5 signaling inhibits regulatory T cell differentiation into Th17 cells [30], [31].

Herein, we demonstrate that curcumin suppresses CD2/CD3/CD28-initiated activation of CD4^+^ T cells at multiple levels. Curcumin not only inhibits CD4^+^ T cell activation, but also induces CD69 up-regulation on CD4^+^ T cells, followed by successive induction of TGF-β production, homing receptor expression and regulatory T cell expansion during late phase activation.

## Materials and Methods

### Ethics Statement

Normal adult blood samples were anonymously provided by the Blood Center of Korean Red Cross, Seoul under the approval of the Institutional Review Board of Korean Red Cross and the agreement for research purpose. The written informed consent from blood donors with respect to taking blood samples for research purposes was obtained and approved by the Ethics Committee of Korean Red Cross. All experimental procedures using human blood were performed under the approval of the Institutional Review Board at the Seoul National University (IRB no. 0806/001–002). Data were all analyzed anonymously.

### Antibodies and Reagents

CD2/CD3/CD28-initiated T cell activation/expansion kit was purchased from Miltenyi Biotec (Auburn, CA, USA), and formulation of anti-CD2/CD3/CD28 antibody-coated beads was performed according to the manufacturer’s instructions. 3-(4,5-Dimethylthiazol-2-yl)-2,5-diphenyltetrazolium bromide (MTT), propidium iodide (PI) and phorbol myristate acetate (PMA) were obtained from Sigma-Aldrich (St Louis, MO, USA). Stock solution of curcumin (Sigma-Aldrich) was prepared in DMSO (Sigma-Aldrich) at 10 mg/mL and stored at −20°C. Curcumin was diluted in fresh media before each experiment, and the final DMSO concentration was lower than 0.08% (v/v). 5,6-carboxyfluorescein diacetate succinimidyl ester (CFSE) was obtained from Invitrogen (Grand Island, NY, USA). Fluorophore-conjugated monoclonal antibodies for surface or intracellular molecules were purchased from BD Bioscience (San Jose, CA, USA), unless otherwise stated, as follows; anti-Annexin-V FITC, -CD25 APC or PE, -CD69 APC or PE, -CD45RO PE, -CD27 FITC, -CCR7 Alexa647, -IL-12RβI PE, -L-selectin PE, -integrin β7 PE, -CD40L PE, -TGF-β PE (R&D Systems, Minneapolis, MN, USA), -IFN-γ APC, -TNF-α PE, -IL-10 PE, -IL-13 PE, -IL-17A APC, -Foxp3 PE antibodies. U0126 (ERK inhibitor), SP600125 (JNK inhibitor), SB203580 (p38 MAPK inhibitor) and a TGF-β receptor I (TGF-βRI) kinase inhibitor were obtained from Calbiochem (San Diego, CA, USA).

### CD4^+^ T Cell Isolation

Peripheral blood mononuclear cells were purified from normal adult human blood by density gradient centrifugation using Ficoll-Paque Plus™ (Amersham Healthcare, Buckinghamshire, UK). CD4^+^ T cells were isolated from peripheral blood mononuclear cells using IMag™ anti-human CD4 antibodies (BD Biosciences). As soon as isolated, the cells were washed with RPMI 1640 more than two times and used immediately for experiments. CD3^+^CD4^+^ T cells are routinely obtained with above 97% purity, verified by flow cytometry during all the experiments. The written informed consent from blood donors with respect to taking blood samples for research purposes was obtained and approved by the Ethics Committee of Korean Red Cross. All experimental procedures using human samples were performed under the approval of the Institutional Review Board at the Seoul National University (IRB no. 0806/001–002).

### Cell Culture and Treatment

CD4^+^ T cells were cultured in RPMI 1640 supplemented with heat-inactivated 10% FBS (Invitrogen) and 1% antibiotics (Invitrogen) at 37°C, 5% CO_2_. The cells were seeded at 2×10^5^ cells/200 µl per well in U-bottomed 96-well plate and activated with anti-CD2/CD3/CD28 antibody-coated beads only (1∶2 or 1∶10 for bead-to-cell ratio) or with either 0.2 or 2 µg/mL of curcumin for the indicated time periods. The un-activated groups were treated with DMSO at 0.08% (v/v). With changing fresh media every 3 days, the cells were extensively washed with PBS, and then transferred to a new culture plate. For the inhibition of MAPK activity, an ERK, JNK or p38 inhibitor (10 µM) was added 1 hour prior to treatment with an additional 2 µg/mL of curcumin after 48 hours of culture. For the inhibition of TGF-β signaling, a TGF-βRI kinase inhibitor (5 µg/mL) was added after 3 days of the culture.

### Proliferation Assay

Before culture, CD4^+^ T cells were labeled with 1 µM of CFSE for 10 min at 37°C in a humidified incubator with 5% CO_2_ in the dark and washed twice with RPMI 1640. Cell proliferation was assessed by flow cytometry (FACSCalibur, BD Bioscience) and FlowJo software (Tree Star, San Carlos, CA, USA). Alternatively, MTT was added at 10% (v/v) concentration into CD4^+^ T cell culture at the indicated time. The cells were incubated for additional 5 hours at 37°C and then centrifuged for 10 min at 400 *g*. The supernatants were discarded, and an equal volume of DMSO was added. Changes in color were detected at 550 nm using a microplate reader (Molecular Devices, Sunnyvale, CA, USA).

### Apoptosis Assay

Cells were harvested and labeled with an anti-Annexin V antibody and PI. After staining for 15 min at 4°C under dark condition, the cells were washed with cold PBS. Cell death was assessed by flow cytometry and FlowJo software.

### Cell Surface and/or Intracellular Staining

Cells were stained with anti-CD25 APC, -CD69 APC or PE, -CD45RO PE, -CD27 FITC, -CCR7 Alexa647, -IL-12RβI PE, -L-selectin PE, -integrin β7 FITC, -CD40L PE and/or -TGF-β PE antibodies for 20 min at 4°C in the dark, and then the cells were washed 3 times with PBS. For intracellular staining, cells were stimulated with PMA (25 ng/mL; Sigma-Aldrich) and ionomycin (250 ng/mL; Sigma-Aldrich) in the presence of Brefeldin A (3 µl/mL; BD Biosciences) at 37°C for 5 hours. Cells were fixed, permeabilized, and stained with anti-IL-2 APC, -IFN-γ APC, -TNF-α PE, -IL-10 PE, -IL-13 PE or -IL-17A APC antibodies for 20 min at 4°C in the dark and washed twice with BD Perm/Wash™ buffer (BD Biosciences). For Foxp3 staining, cells were stained with anti-Foxp3 PE antibody using a human Foxp3 staining buffer set (BD Biosciences) according to the manufacturer’s instructions. All samples were analyzed by flow cytometry and FlowJo software.

### Cytokine Assay

The levels of IL-2, IL-10, IFN-γ and active TGF-β1 in culture supernatants were quantified by ELISA DuoSet (R&D Systems) according to the manufacturer’s instructions. For detection of active TGF-β1, the samples were processed by oxidation-reduction according to the manufacturer’s instructions.

### Functional Assay

To test a regulatory activity of curcumin-treated CD4^+^ T cells, curcumin-treated and activated CD4^+^ cells were co-cultured with autologous CD4^+^ T cells with or without CD2/CD3/CD28 stimulation for 3 days. Prior to co-culture, CD4^+^ T cells were activated with anti-CD2/CD3/CD28 antibody-coated beads (1∶10 for bead-to-cell ratio) with or without 2 µg/mL of curcumin for 3 days and autologous CD4^+^ T cells were maintained in the fresh media containing 2.5 ng/ml of hrIL-2 (R&D Systems, Minneapolis, MN, USA) for 3 days [32] followed by CFSE labeling. Before the co-culture, all the cells were washed with PBS.

### Statistical Analysis

The mean ± SD was determined on the basis of 3 different blood samples. All the experiments were performed at least three times with different blood samples. All the data are representative of independent experiments yielding similar results. Each treatment group was compared with the appropriate control group, and statistical significance was measured using a two-tailed paired *t*-test. Differences were considered significant when *P*<0.05. Prism 5 software (GraphPad Software Inc., San Diego, CA, USA) was used to perform all statistical analyses and to generate graphs.

## Results

### Curcumin Inhibits CD2/CD3/CD28-initiated CD4^+^ T Cell Proliferation

**Figure 1:**
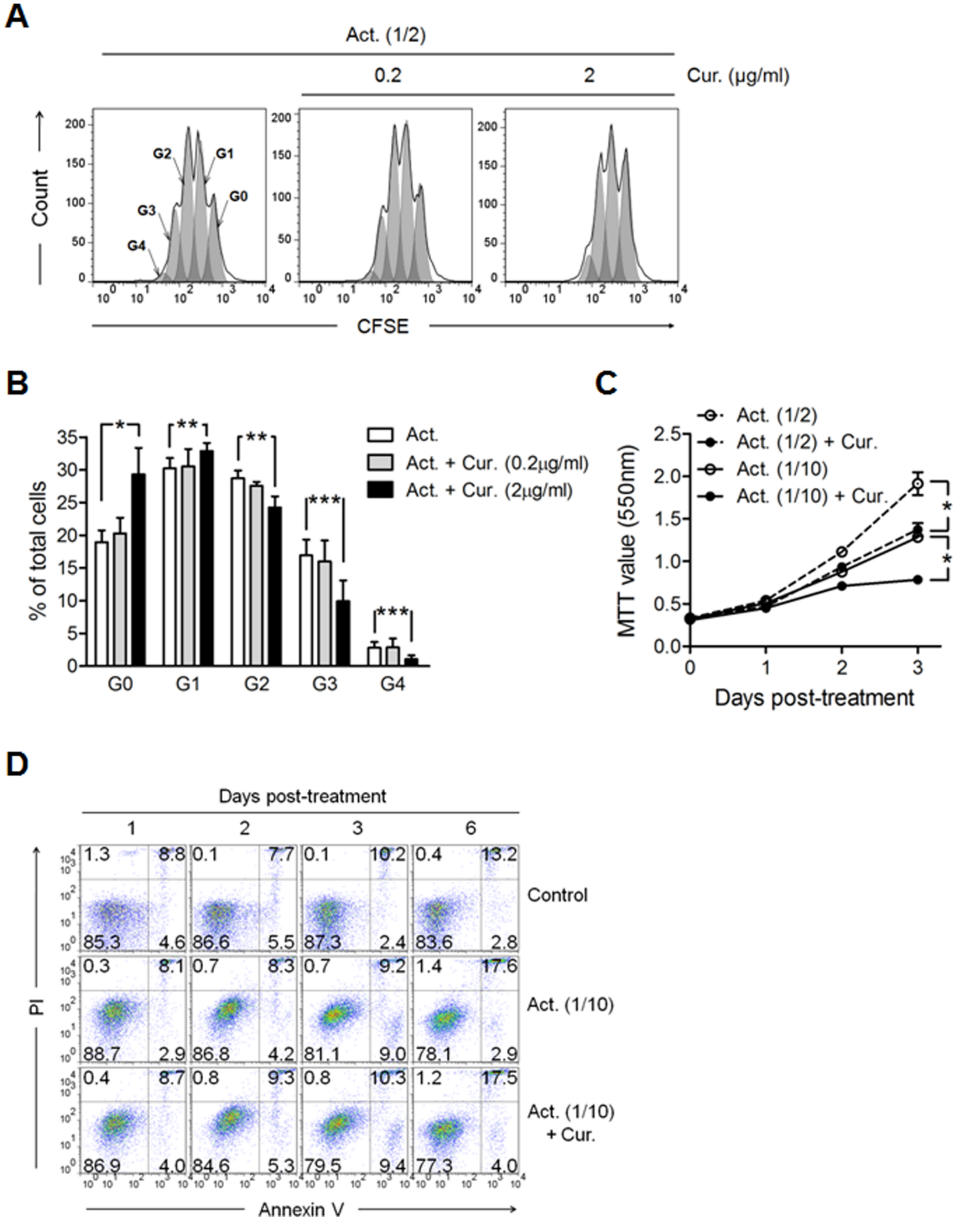
Curcumin inhibits CD4^+^ T cell expansion induced by CD2/CD3/CD28 signaling without inducing cell death. CD4^+^ T cells were cultured in the presence of anti-CD2/CD3/CD28 antibody-coated beads only (Act.) or with either 0.2 or 2 µg/mL of curcumin (Cur.) for the indicated time periods. Act. (1/2) and Act. (1/10) represent bead-to-cell ratios of 1∶2 and 1∶10, respectively. (A, B) For cell proliferation assay, the cells were labeled with CFSE prior to culture, and harvested at 3 days of culture. (A) Cell generations (G0∼G4) were calculated and (B) plotted as the percentage of total cells by using flow cytometry and FlowJo software. (C) Results of an MTT cell proliferation assay to assess cell numbers at 1, 2 and 3 days of culture. (D) Cells were harvested at the indicated time points and labeled with an anti-Annexin V antibody and PI. The numbers in the plot indicate the percentage of cells in the respective areas. Data are representative of 3 experiments yielding similar results. (C and D) Curcumin was added at a concentration of 2 µg/mL. (B and C) Data are presented as the mean ± SD. *P<0.05, **P<0.01, ***P<0.001.

We initially determined the impact of different concentrations of curcumin on human primary CD4^+^ T cells. Results indicated that CD4^+^ T cell death sharply increased when the cells were treated with 20 µg/mL of curcumin for 24 h, but that there was no change in cell viability following treatment with 0.2 or 2 µg/mL of curcumin (Figure S1). Based on these results, 20 µg/mL of curcumin is toxic to CD4^+^ T cells and this concentration was not used in subsequent experiments. In order to investigate the current controversy regarding the effect of curcumin on T cell activity [7]–[11], [14]–[16], we tested whether curcumin dosed at 0.2 or 2 µg/mL modulated CD2/CD3/CD28-initiated CD4^+^ T cell proliferation. CD4^+^ T cells labeled with CFSE were stimulated with anti-CD2/CD3/CD28 Abs to induce proliferation, and then treated with curcumin for 3 days. Treatment with curcumin at 2 µg/mL increased the percentage of zero (G0) and the 1^st^ generation (G1) T cells, but decreased the generations from the 2^nd^ (G2) to the 4^th^ (G4) activated T cells, indicating that curcumin inhibits CD4^+^ T cell expansion (Figure 1A and 1B). This finding was further confirmed using an MTT proliferation assay (Figure 1C). Collectively, these results suggest that curcumin has a negative impact on CD4^+^ T cell proliferation, regardless of the degree of CD2/CD3/CD28 signaling.

We also evaluated whether curcumin induces apoptosis in CD2/CD3/CD28-activated CD4^+^ T cells. Treatment of CD4^+^ T cells with curcumin at 2 µg/mL did not induce apoptotic cell death under conditions of CD2/CD3/CD28 stimulation (Figure 1D), reducing the likelihood that lower cell counts were caused by cell death (Figure 1C). These results suggested that 2 µg/mL of curcumin was a valid concentration for use in further experiments.

### Curcumin Inhibits CD2/CD3/CD28-mediated CD4^+^ T Cell Activation

**Figure 2:**
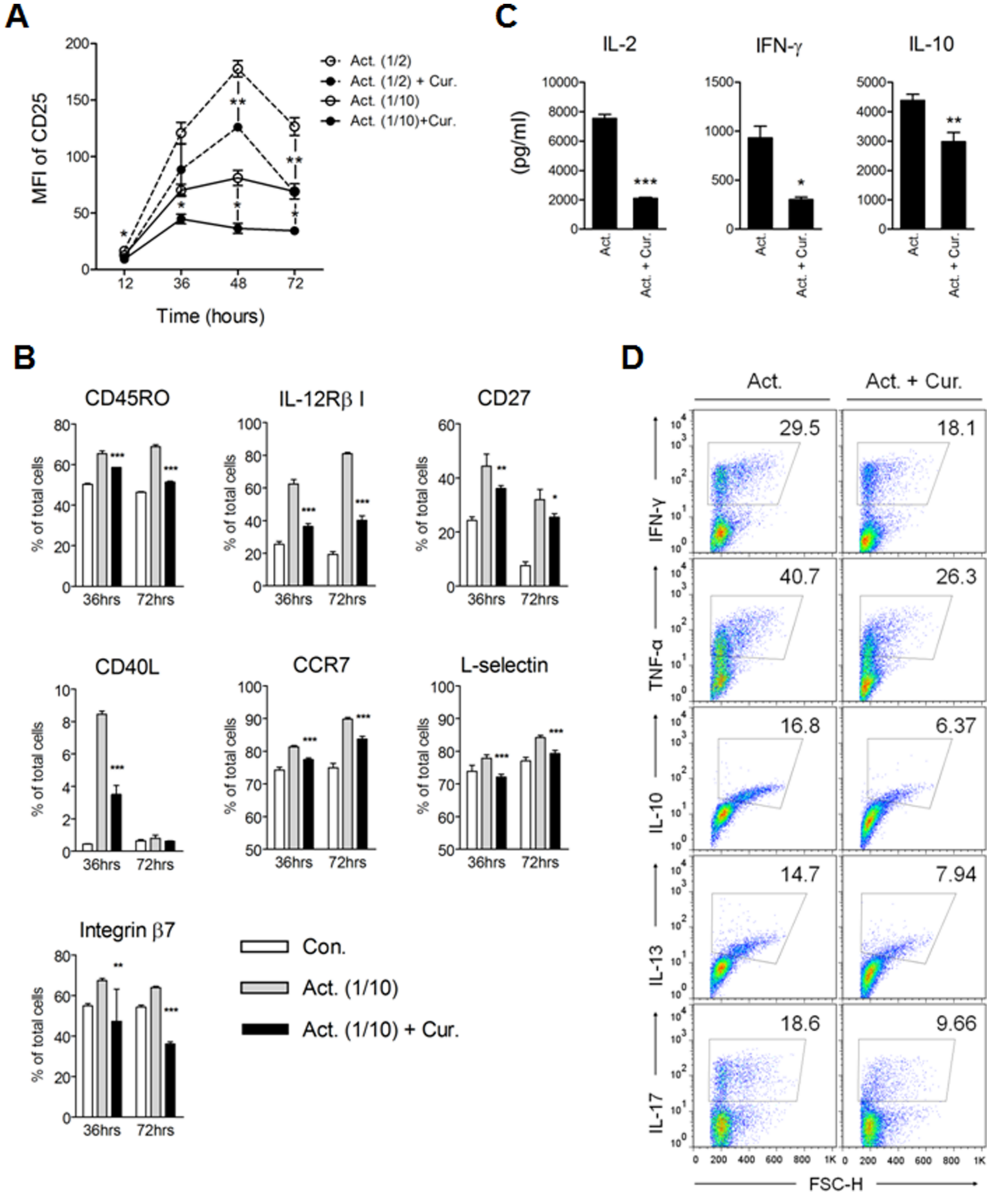
Curcumin suppresses CD4^+^ T cell activation and differentiation. CD4^+^ T cells were cultured in the presence of anti-CD2/CD3/CD28 antibody-coated beads alone (Act.) or with 2 µg/mL curcumin (Cur.) for 3 days. Act. (1/2) and Act. (1/10) indicate a 1∶2 and 1∶10 bead-to-cell ratio, respectively. Cells were harvested at the indicated time points and the expression of (A) CD25 and (B) CD45RO, IL-12Rβ1, CD27, CD40L, CCR7, L-selectin and Integrin β7 was determined by flow cytometric analysis. (A) Mean fluorescence index (MFI) and (B) percentage of the cells expressing each molecule was calculated by using FlowJo software. (C) The supernatants were collected at 3 days of culture and the total cytokine level was determined using sandwich ELISA. (D) After 3 days of culture, cells were collected, extensively washed with PBS, and then transferred to new cell culture plate in fresh media for an additional 3 days. Following culture, the cells were stimulated with PMA and ionomycin plus Brefeldin A for an additional 5 hours, and then intracellular cytokine content was evaluated. The numbers in plots indicate the percentage of cytokine-expressing cells. Results are representative of 3 replicate experiments yielding similar results. (A, B and C) Data are presented as the mean ± SD. *P<0.05, **P<0.01, ***P<0.001.

We then assessed whether curcumin suppresses CD2/CD3/CD28-initiated activation of CD4^+^ T cells. We found that without CD2/CD3/CD28 stimulation, 2 µg/mL of curcumin had no effect on CD25 (IL-2 receptor α), CD69 and CD45RO expressions on CD4^+^ T cells (data not shown). Conversely, treatment with curcumin significantly (*P*<0.05–0.01) reduced CD2/CD3/CD28-initiated CD25 expression (Figure 2A). Coinciding with down-regulated CD25 expression, expression of the molecules responsible for T cell function was also decreased (Figure 2B) and, as expected, CD2/CD3/CD28-initiated cytokine production was also significantly (*P*<0.05–0.001) reduced (Figure 2C). To determine whether decreased cytokine levels were an immediate consequence of curcumin treatment (Figure 1C), we examined intracellular levels of the cytokines IL-2, IFN-γ, TNF-α, IL-10 and IL-13 in CD4^+^ T cells. Intracellular levels of the cytokines produced by CD2/CD3/CD28 signaling were reduced following curcumin treatment (Figure S2), suggesting that curcumin directly inhibits CD2/CD3/CD28-initiated cytokine production in CD4^+^ T cells.

Next, we investigated whether the immunosuppressive effects of curcumin on CD4^+^ T cell activation was still maintained after resting. After 3 days of culture with CD2/CD3/CD28 stimulation with or without curcumin, CD4^+^ T cells were harvested, washed, transferred to a new cell culture plate, and cultured in fresh media for an additional 3 days to allow any residual CD2/CD3/CD28 stimulus to end while CD2/CD3/CD28-mediated activation was fulfilled. After a total of 6 days in culture, the cells were re-stimulated with PMA and ionomycin for an additional 5 hours and intracellular cytokine profiles were monitored by flow cytometry. CD4^+^ T cells treated with curcumin exhibited lower percentages of Th1 (IFN-γ and TNF-α), Th2 (IL-10 and IL-13), or Th17 (IL-17) cytokine-positive cells than those without curcumin treatment, indicating that curcumin’s inhibitory effects on CD4^+^ T cell activity was still maintained even after an extensive resting (Figure 2D).

### Curcumin Attenuates the Decline of CD69 Expression and Up-regulates CCR7 and L-selectin Expression at Late Phase

**Figure 3:**
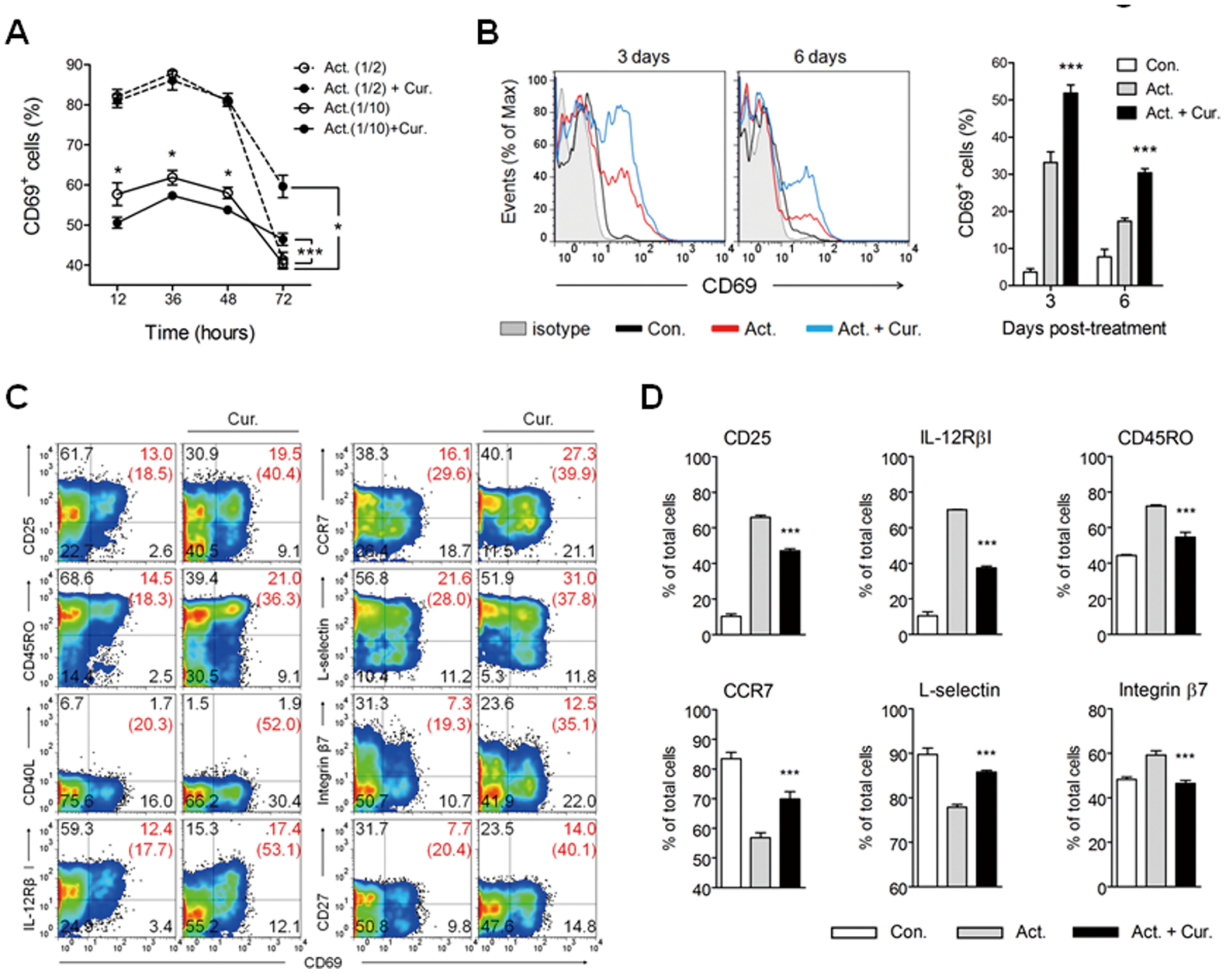
Curcumin attenuates late-phase CD69 decline and up-regulates late-phase CCR7 and L-selectin expression. CD4^+^ T cells were cultured in the presence of anti-CD2/CD3/CD28 antibody-coated beads only (Act.) or with 2 µg/mL curcumin (Cur.) for 3 days. Act. (1/2) and Act. (1/10) indicate a 1∶2 and 1∶10 bead-to-cell ratio, respectively. (A) Cells were harvested at the indicated time point and the percentage of CD69^+^ cells was determined by flow cytometric analysis. (B–D) After 3 days of culture, cells were harvested, washed with PBS, and then transferred to a new cell culture plate in fresh media for an additional 3 days. The cells were then stained and analyzed by flow cytometry. (B) Histograms of CD69 expression and the total percentage of CD69^+^ cells. (C) The numbers in each plot and the number in blanket indicate the percentage of cells in each respective area and the percentage of CD69^+^ cells among cells positive with the Y axis, respectively. (D) The percentage of total cells positive for each molecule. Data are representative of 3 replicate experiments yielding similar results. (A, B and D) Data are presented as the mean ± SD. *P<0.05, ***P<0.001.

Similar to the other molecules (Figure 2A and B), CD2/CD3/CD28-mediated induction of CD69^+^ cells was also inhibited by curcumin treatment for 48 hours (Figure 3A). However, the percentage of CD69^+^ cells was higher in the curcumin treatment groups than in groups of cells not at late phase when CD69 expression had spontaneously decreased. It is important to note that the CD69 down-regulation with curcumin treatment disappeared during strong CD2/CD3/CD28 stimulation (1∶2 for bead to cell ratio), whereas the CD69 up-regulation was observed regardless of the strength of CD2/CD3/CD28 signaling at late phase (e.g., from day 4–6 culture) (Figure 3A and 3B). Indeed, curcumin increased the percentage of cells that are double positive for CD69 plus other cell surface molecules, with the exception of CD40L (Figure 3C). As a result, curcumin markedly enhanced the percentage of CD69^+^ cells among the populations positive with each effector molecule whose expression was decreased by curcumin (the number in the blanket of Figure 3C).

Moreover, it is important to note that the expression of CCR7 and L-selectin was up-regulated by curcumin at late phase (e.g., 6-day culture) (Figure 3D), whereas expression was decreased following 3 days in culture (Figure 2B). This pattern was not observed for integrin β7, implying that curcumin indirectly but selectively up-regulates the expression of the homing receptors CCR7 and L-selectin at late-phase.

### ERK_1/2_ Signal Pathway is Involved in Late-phase Expression of CD69 by Curcumin

**Figure 4:**
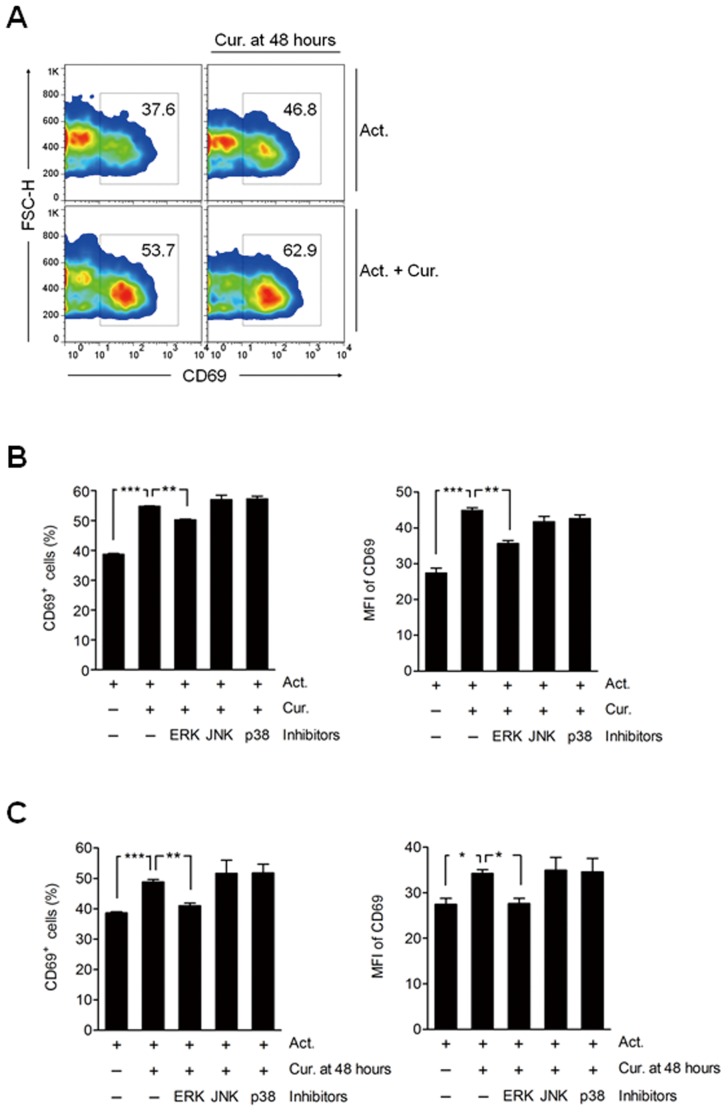
ERK_1/2_ involvement in curcumin-mediated up-regulation of late-phase CD69 expression. (A, B) CD4^+^ T cells were cultured in the presence of anti-CD2/CD3/CD28 antibody-coated beads only (Act.; 1∶10 for bead to cell) or with 2 µg/mL curcumin (Cur.) for 3 days. Cells were then harvested and the percentage of CD69^+^ cells was determined by flow cytometric analysis. (A) After 48 hours of culture, cells were treated with an additional 2 µg/mL of curcumin (Cur. 48 hours). The number in each panel indicates the total percentage of CD69^+^ cells. The results are the representative of 3 replicate experiments yielding similar results. (B) After 48 hours of culture, cells were treated with 10 µM of U0126 (ERK inhibitor), SP600125 (JNK inhibitor), or SB203580 (p38 MAPK inhibitor). (C) CD4^+^ T cells were cultured in the presence of anti-CD2/CD3/CD28 antibody-coated beads (Act.; 1∶10 for bead-to-cell ratio) for 3 days, with 10 µM of U0126 (ERK inhibitor), SP600125 (JNK inhibitor), or SB203580 (p38 MAPK inhibitor) added 1 hour prior to treatment with an additional 2 µg/mL of curcumin (Cur. 48hrs) after 48 hours of culture. CD69 expression was assessed by flow cytometry. Data are presented as the mean ± SD. **P<0.01, ***P<0.001.

Next, we evaluated whether curcumin treated at later time point induces CD69 expression on activated CD4^+^ T cells. Interestingly, late CD69 expression was increased in both groups, activated together with curcumin-treated (Act.+Cur.) and activated (Act.) followed by post-treatment with curcumin (Cur. at 48 hours) (Figure 4A). This result indicates that curcumin treatment at distinct time points of T cell activation has same effects on the late CD69 expression.

Increased ERK_1/2_ activation has been observed in CD69^+^ CD4^+^ T cells, recently characterized as a novel subset of regulatory T cells both in human and mouse [33], [34]. Besides, it is recently reported that early and late expression of CD69 on activated T lymphocytes are differently controlled by ERK_1/2_ downstream transcriptional factors through canonical and non-canonical NF-κB signalings [35]. Thus, we examined whether the late up-regulation of CD69 by curcumin was solely and directly due to increased ERK_1/2_ activation. Curcumin treatment at either early or late time point decreased late ERK_1/2_ activation in the activated T cells (Figure S3). Interestingly, higher ERK_1/2_ activation in CD69^+^ than that in CD69^−^ T cells upon curcumin treatment remained the same (data not shown). These results suggest that ERK_1/2_ activation is not an exclusive factor for the late induction of CD69. Instead, we further found that the up-regulation of CD69 by curcumin was significantly (*P*<0.01) decreased by treatment with an ERK inhibitor, but not in the presence of a JNK or p38 inhibitor (Figure 4B). When curcumin was added at 48 hours of T cell activation following a 1 hour pre-inhibition with an ERK, JNK or p38 inhibitor, CD69 up-regulation was almost completely inhibited by the ERK inhibitor (Figure 4C), suggesting that the ERK_1/2_-dependent signal pathway is still involved in the late CD69 expression upon curcumin treatment.

### TGF-β1 Influences Curcumin-mediated Suppression of CD4^+^ T Cell Activation

**Figure 5:**
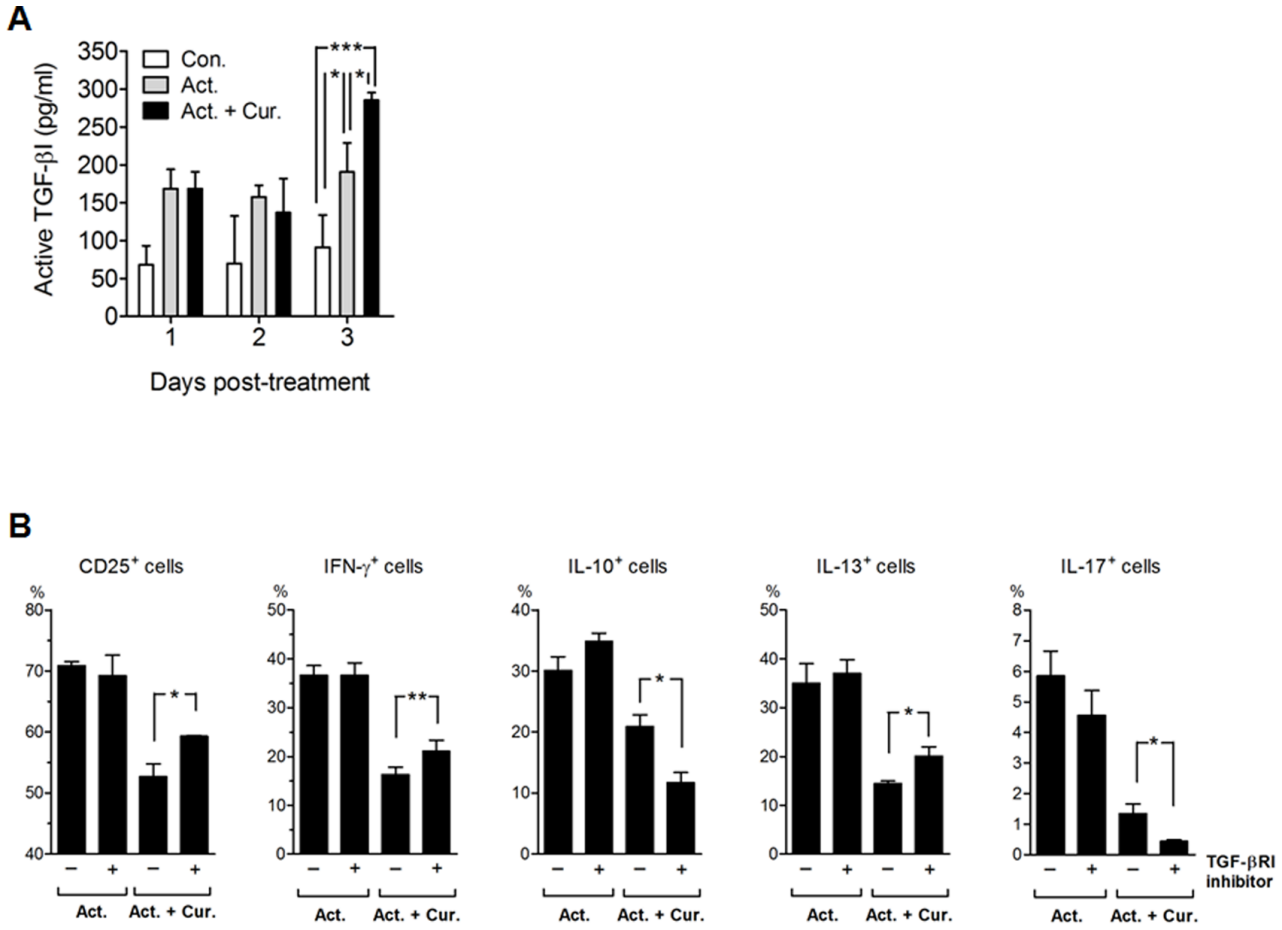
The influence of TGF-β1 on the regulation of CD4^+^ T cell activation following curcumin treatment. CD4^+^ T cells were cultured in the presence of anti-CD2/CD3/CD28 antibody-coated beads only (Act.; 1∶10 for bead-to-cell ratio) or with 2 µg/mL of curcumin (Cur.) for 3 days. (A) Cell culture supernatants were collected at the indicated time point and total levels of active TGF-β were determined using an ELISA. (B) After 3 days of culture, cells were collected, washed with PBS and then transferred to a new cell culture plate in order to re-culture the cells in fresh media with or without a TGF-βRI kinase inhibitor (5 µg/mL) for an additional 3 days. The percentage of CD25^+^, IFN-γ^+^, IL-10^+^, IL-13^+^ and IL-17^+^ cells was determined by flow cytometric analysis. For detection of cytokine-producing cells, the cells were re-stimulated with PMA and ionomycin plus Brefeldin A for 5 hours. Data are presented as the mean ± SD. *P<0.05, **P<0.01, ***P<0.001.

We next determined whether TGF-β1 is produced subsequent to the attenuated decline of CD69 expression. Results indicated that the production of active TGF-β1 at 3 days of culture was increased by curcumin treatment (Figure 5A). The increase in TGF-β1 expression was moderate, but distinct, in light of the fact that the total number of cells was significantly decreased by curcumin (Figure 1C).

We then tested whether the increased production of TGF-β1 influences curcumin regulation of CD4^+^ T cell activation. The reduced percentage of cells positive for CD25, or producing IFN-γ or IL-13 after curcumin treatment was partially recovered by cell exposure to a TGF-β RI kinase inhibitor, concurrent with a dramatic decrease in IL-10 or IL-17 - producing cells (Figure 5B). These results suggest that TGF-β1 plays a unique role in regulating T cell activation at late phase after curcumin treatment.

### TGF-β1 Involvement in Curcumin-mediated Regulatory T Cell Generation at Late Phase

**Figure 6:**
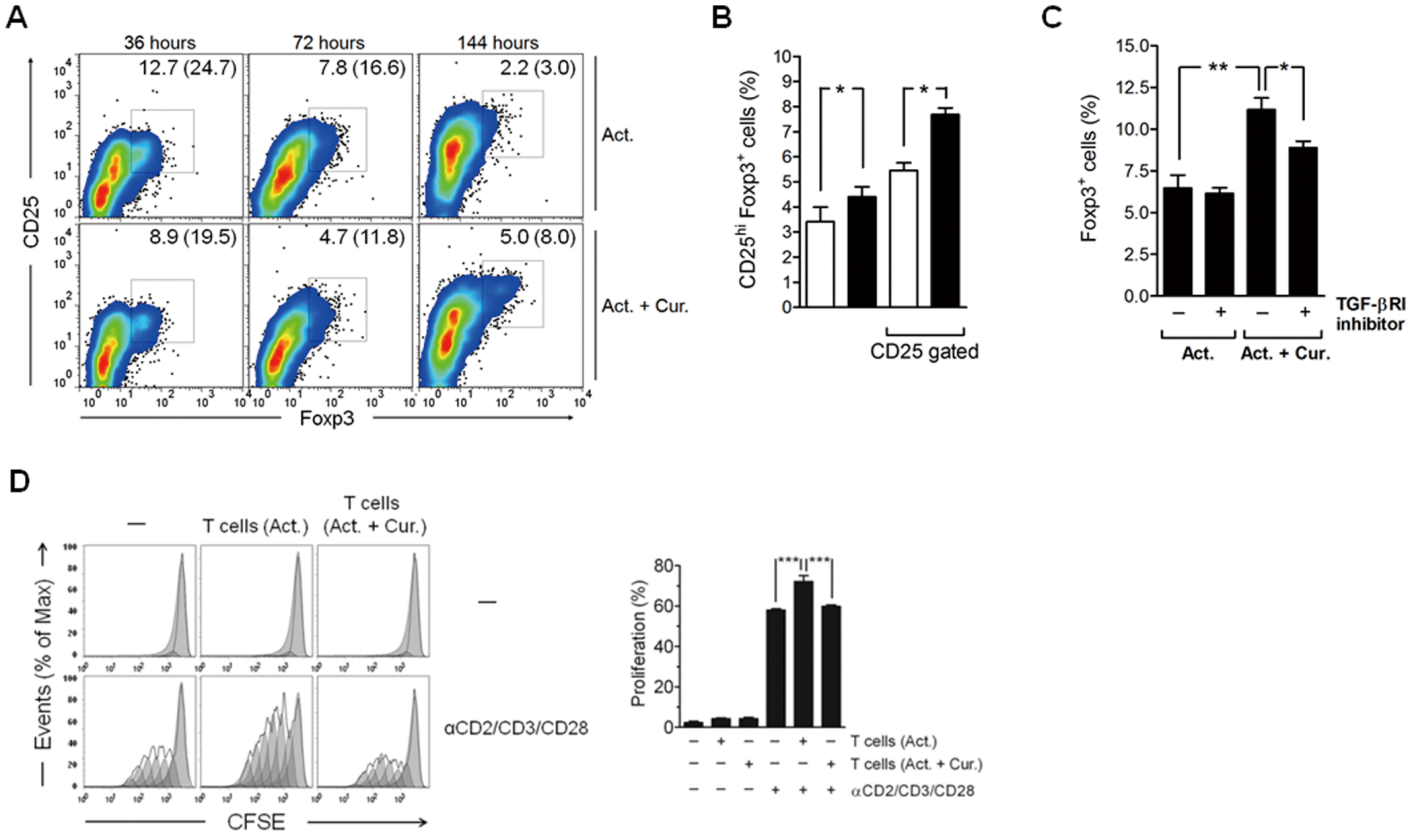
TGF-β1 is associated with curcumin-mediated generation of regulatory T cells at late phase. CD4^+^ T cells were cultured in the presence of anti-CD2/CD3/CD28 antibody-coated beads only (1∶10 for bead-to-cell ratio) or with 2 µg/mL of curcumin (Cur.) for 3 days, and then transferred to a new cell culture plate and incubated with a fresh media for an additional 3 days. (A) Cells were collected at the indicated time points and labeled with anti-CD25 and anti-Foxp3 antibodies. The cells were then washed and analyzed for CD25 and Foxp3 expression by flow cytometry. The numbers in each panel and the numbers in blankets indicate the percentage of CD25^hi^Foxp3^+^ cells in total and among CD25^+^ cells, respectively. (B) After 6 days of culture, cells were collected and percentage of CD25^hi^Foxp3^+^ cells in total and among CD25^+^cells was determined by flow cytometric analysis. The empty and filled bars indicate cells treated with beads only and cells treated with both beads and curcumin, respectively. (C) A TGF-βRI kinase inhibitor (5 µg/mL) was added after 3 days of culture. The percentage of Foxp3^+^ cells was determined by flow cytometry. (D) After 3 days of culture, the cells were washed with PBS and then co-cultured with CFSE-labeled autologous CD4^+^ T cells with or without CD2/CD3/CD28 stimulation for 3 days. The cell proliferation was determined by flow cytometric analysis. (B, C and D) Data are presented as the mean ± SD. *P<0.05, **P<0.01, ***P<0.001.

Interestingly, we also found that, in contrast to results from earlier time points, the percentage of CD25^hi^Foxp3^+^ regulatory T cells was higher after 6 days of culture with curcumin than in cells without curcumin treatment (Figure 6A and 6B), suggesting that curcumin indirectly induces late generation of regulatory T cells following CD2/CD3/CD28 activation. The generation of regulatory T cells at late phase was partially diminished by the addition of a TGF-βRI kinase inhibitor (Figure 6C), suggesting that the increased TGF-β1 production is responsible, at least partially, for the late emergence of regulatory T cells by curcumin treatment.

Furthermore, curcumin-treated and activated CD4^+^ T cells had a regulatory activity against autologous CD4^+^ T cells. CD2/CD3/CD28-mediated proliferation of autologous CD4^+^ T cells was not increased by co-culture of curcumin-treated and activated CD4^+^ T cells, whereas it was increased by only activated one (Figure 6D). In addition, cell surface molecule (CD40, CD80 and CD83) expression level on CD11c^+^ DCs when co-cultured with curcumin-treated and activated T cells was inferior to those without curcumin treatment (Figure S4). These regulatory effects of curcumin-treated CD4^+^ T cells indirectly demonstrated the late generation of regulatory T cells by curcumin treatment.

## Discussion

In this study, curcumin not only suppressed CD4^+^ T cell activation and differentiation, but also differently regulated CD69, CCR7 and L-selectin expression, depending on the specific phase of T cell activation. In addition, Curcumin indirectly induced late-phase generation of regulatory T cells, in which TGF-β1 signaling is partially involved, suggesting that curcumin regulates CD4^+^ T cells at multiple levels.

We found that curcumin attenuates spontaneous decline of CD69 expression on activated CD4^+^ T cells. It is noteworthy that CD4^+^CD69^+^ T cells exist persistently within infiltrates of chronic inflammatory sites such as the rheumatic arthritic synovium [28], [29], [36]. At these sites, peripheral CD4^+^ T cells are alternatively activated in an antigen-independent manner by interaction with adhesion receptors and/or constant stimulation by different cytokines at lower concentrations than are required for full T cell activation. Meanwhile, CD69 expression on activated T cells is sustained by co-culture with mesenchymal stem cells *in vitro* [35]. Mesenchymal stem cells are also well known for their potentials to suppress proliferation of activated T cells and induce regulatory T cells by secreting immunoregulatory factors such as prostaglandin E2, indoleamine 2,3-dioxygenase, and TGF-β1 [37]–[39]. Consider these reports, it is conceivable that curcumin-treated CD4^+^ T cells are put under microenvironment closely akin to where T cell-derived cytokines such as IL-2 are absent yet treated with immunomodulator(s) enough to induce alternative activation and regulation.

Previous studies have shown that Erg-1, Erg-3, ATF-3/CREB and AP-1 cooperatively control CD2/CD3/CD28-initiated CD69 expression on CD4^+^ T cells by interacting with *cis*-acting elements within the CD69 promoter [40], [41]. Especially, the interaction of vav with Ras/MEK/ERK signaling leads to up-regulation of CD69 expression, which is known to be dominant during CD2/CD3/CD28-initiated expression [42], [43]. This coincides with recent findings in that CD69^+^ LAP^+^ CD4^+^ T cells, a novel regulatory T cell subset, showed increased ERK_1/2_ activation [33], [34]. In addition, canonical NF-κB signaling promotes early CD69 expression but inhibits its later sustained expression on activated T cells, whereas non-canonical NF-κB signaling promotes the late and sustained CD69 expression [35]. It was further noting that inhibition and replacement of canonical NF-κB signaling by non-canonical signaling correlates with sustained CD69 expression at late phase of T cell activation [35]. We found that curcumin decreases CD2/CD3/CD28-triggered ERK1/2 activation in CD4^+^ T cells at both of early and late phases and that the later sustained CD69 expression by curcumin treatment is diminished by inhibition of ERK_1/2_ activation, suggesting that the ERK_1/2_ pathway is dominant but not exclusive in curcumin up-regulation of late CD69 expression. Given the unique roles played by ERK_1/2_-downstream NF-κB signaling in CD69 expression [35], we could expect that inhibition of late ERK_1/2_ activation by curcumin treatment would induce suppression of canonical NF-κB signaling negative to sustained CD69 expression at later time point; it is reasonable to assume that curcumin might trigger the shift in NF-κB pathways, which promotes attenuated decline of late CD69 expression.

CD69 is known to play a unique role in lymphocyte migration. CD69 inhibits the function of S1P_1_, required for lymphocyte egress from lymph nodes [26], [27]. S1P_1_ is thought to promote lymphocyte egress by overcoming CCR7-mediated retention signals [44]. Integrin β7 is responsible for extravasation of lymphocytes from the blood to diffuse into gut epithelial and lamina propria effector sites, whereas CCR7 and L-selectin (homing receptor) promote immune cell emigration from peripheral tissues into lymphoid tissues [45]–[47] The present results showed that curcumin indirectly induces CCR7 and L-selectin expression at 6 days of culture. Together with the fact that CCR7 and L-selectin expression are reduced by curcumin treatment until 3 days of culture, it might be expected that late-phase regulation of CD69 expression by curcumin might be involved in CCR7 and L-selectin up-regulation. Moreover, increases in the expression of CCR7 and L-selectin, but not integrin β7, are in accordance with previous reports that CD69 plays an critical role in attenuating the accumulation of inflammatory lymphocytes in peripheral non-lymphoid tissues [23], [26], [27].

We observed increased TGF-β1 production subsequent to an attenuation of CD69 decline with curcumin treatment. TGF-β1 production is partially responsible for the regulation of CD4^+^ T cell activation following curcumin treatment through decreases in CD25^+^ cells, IFN-γ^+^ cells and IL-13^+^ cells, and induction of IL-10^+^ cells and IL-17^+^ cells. In addition, TGF-β1 is involved with late-phase generation of regulatory T cells. These observations suggest that the amount of TGF-β1 was sufficient to show an anti-inflammatory effect and to drive production of IL-10 and IL-17 by T cells. A reduced level of TGF-β1 plays a major role in exacerbating the severity of arthritis in a CD69-deficient mouse model [22]. The engagement of an anti-CD69 antibody with CD3^+^ T cells leads to the production of TGF-β1. Thus, our results suggested that the late CD69 up-regulation with curcumin treatment might be one of the major factors potentiating TGF-β1 production.

Curcumin has been intensively studied as a promising therapeutic agent for the treatment of chronic inflammatory diseases such as arthritis, allergy and asthma [17], [20], [48]. Although CD4^+^ T cells have been implicated in the pathogenesis of chronic inflammation [21], the effects of curcumin on the regulation of peripheral CD4^+^ T cells have not been elucidated in detail, but might be associated with these diseases. Hence, it is intriguing that concomitant with up-regulation of the late-phase expression of CD69, curcumin augments the expression of homing receptors and TGF-β1 followed by the generation of regulatory T cells, implying that curcumin might relieve local inflammatory stress by directly dampening T cell-driven inflammation at multiple levels. The present study offers one of potential, but perhaps crucial, mechanism in which curcumin could alleviate chronic inflammatory diseases through CD4^+^ T cell-based therapeutic approach.

## Supporting Information

Figure S1 **The impact of different concentrations of curcumin on viability of human primary CD4^+^ T cells.** CD4^+^ T cells were cultured with curcumin (Cur., 0.2, 2 or 20 µg/mL) or without curcumin for 1 day, labeled with an anti-Annexin V antibody and PI, and then analyzed by flow cytometry. The numbers in panel indicate the percentage of cells in the respective area.
(TIF)

Figure S2 **Curcumin directly inhibits CD2/CD3/CD28-initiated cytokine production in CD4^+^ T cells.** CD4^+^ T cells were cultured in the presence of anti-CD2/CD3/CD28 antibody-coated beads only (Act.) or with 2 µg/mL of curcumin (Cur.) using a 1∶10 bead-to-cell ratio. Cells were treated with Brefeldin A (3 µl/mL) for 5 hours prior to harvest at 1 days of culture, and then harvested, labeled and analyzed by flow cytometry.
(TIF)

Figure S3 **Curcumin decreases ERK_1/2_ activation in CD2/CD3/CD28-activated CD4^+^ T cells.** CD4^+^ T cells were cultured in the presence of anti-CD2/CD3/CD28 antibody-coated beads (Act., 1∶10 for bead-to-cell ratio) only or with curcumin treatment (2 µg/mL) at the beginning of culture (Cur.) or at 48 hours of culture (Cur. at 48 hours). After total 3 days culture, the cells were fixed, permeabilized, and stained with anti-ERK1/2 (pT202/pY204) PE antibody (BD Phosflow), and then analyzed by flow cytometry.
(TIF)

Figure S4 **The regulatory effect of CD4^+^ T cells treated with curcumin and co-cultured with DCs.** CD4^+^ cells were activated with anti-CD2/CD3/CD28 antibody-coated beads (1∶10 for bead-to-cell ratio) with/without 2 µg/mL of curcumin for 5 days with changing fresh media every 3 days, and then co-cultured with DCs for additional 1 day. Autologous DCs were derived from human CD14^+^ monocyte and treated with IL-4 and GM-CSF for 5 days. Before the co-culture, CD4^+^ T cells and DCs were washed with PBS. The expression of CD40, CD80 and CD83 on CD11c^+^ DCs were determined by flow cytometric analysis.
(TIF)
