# Correction: Curcumin Inhibits CD4^+^ T Cell Activation, but Augments CD69 Expression and TGF-β1-Mediated Generation of Regulatory T Cells at Late Phase

**DOI:** 10.1371/annotation/631b0f02-bf10-4bac-88a3-c986f2b73284

**Published:** 2013-05-23

**Authors:** Girak Kim, Mi Seon Jang, Young Min Son, Min Ji Seo, Sang Yun Ji, Seung Hyun Han, In Duk Jung, Yeong-Min Park, Hyun Jung Jung, Cheol-Heui Yun

There was an error in the affiliation for author Cheol-Heui Yun. The affiliations for Cheol-Heui Yun are as follows:

1 Department of Agricultural Biotechnology and Research Institute for Agriculture and Life Sciences, Seoul National University, Seoul, Republic of Korea

5 Center for Food and Bioconvergence, Seoul National University, Seoul, Republic of Korea

6 World Class University Biomodulation Major, Department of Agricultural Biotechnology, Seoul National University, Seoul, Republic of Korea

There was an error in the funding information. The correct funding statement is:

This work was supported by Basic Science Research Program through the National Research Foundation (NRF) (2010-0027222, 2010-0003291, World Class University program (R31-10056)) and a grant from the Next-Generation BioGreen 21 Program (PJ008127012011), Rural Development Administration and by R&D Convergence Center Support Program, Ministry for Food, Agriculture, Forestry and Fisheries, Republic of Korea. The funders had no role in study design, data collection and analysis, decision to publish, or preparation of the manuscript.

