# Supplementary figures and images for: Curcumin Inhibits CD4+ T Cell Activation, but Augments CD69 Expression and TGF-β1-Mediated Generation of Regulatory T Cells at Late Phase
Source: PLoS One. 2013 Apr 26;8(4):e62300. doi: 10.1371/journal.pone.0062300 (PMC3637266; doi:10.1371/journal.pone.0062300)

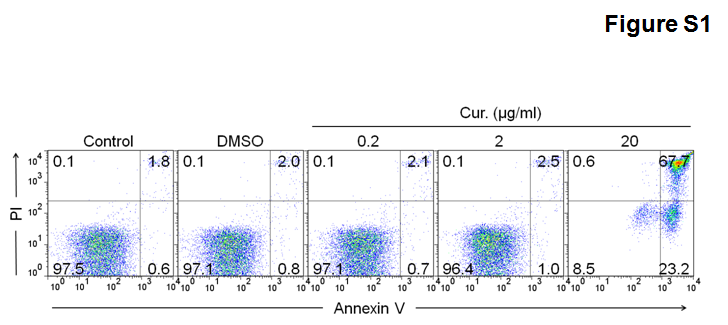

Supplement: Figure S1 — The impact of different concentrations of curcumin on viability of human primary CD4+ T cells. CD4+ T cells were cultured with curcumin (Cur., 0.2, 2 or 20 µg/mL) or without curcumin for 1 day, labeled with an anti-Annexin V antibody and PI, and then analyzed by flow cytometry. The numbers in panel indicate the percentage of cells in the respective area. (TIF) [file pone.0062300.s001.tif]

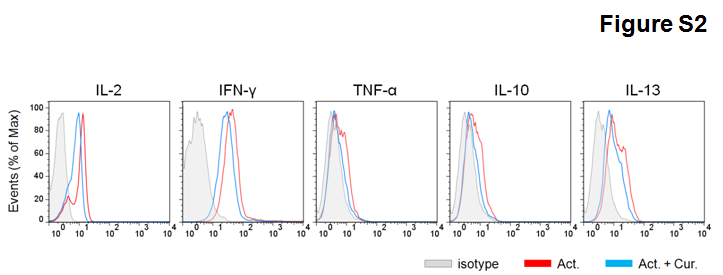

Supplement: Figure S2 — Curcumin directly inhibits CD2/CD3/CD28-initiated cytokine production in CD4+ T cells. CD4+ T cells were cultured in the presence of anti-CD2/CD3/CD28 antibody-coated beads only (Act.) or with 2 µg/mL of curcumin (Cur.) using a 1∶10 bead-to-cell ratio. Cells were treated with Brefeldin A (3 µl/mL) for 5 hours prior to harvest at 1 days of culture, and then harvested, labeled and analyzed by flow cytometry. (TIF) [file pone.0062300.s002.tif]

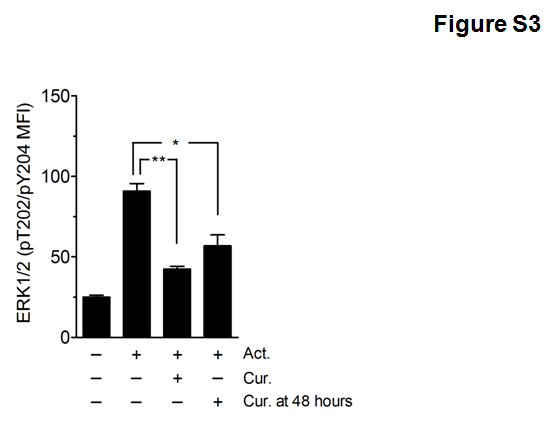

Supplement: Figure S3 — Curcumin decreases ERK1/2 activation in CD2/CD3/CD28-activated CD4+ T cells. CD4+ T cells were cultured in the presence of anti-CD2/CD3/CD28 antibody-coated beads (Act., 1∶10 for bead-to-cell ratio) only or with curcumin treatment (2 µg/mL) at the beginning of culture (Cur.) or at 48 hours of culture (Cur. at 48 hours). After total 3 days culture, the cells were fixed, permeabilized, and stained with anti-ERK1/2 (pT202/pY204) PE antibody (BD Phosflow), and then analyzed by flow cytometry. (TIF) [file pone.0062300.s003.tif]

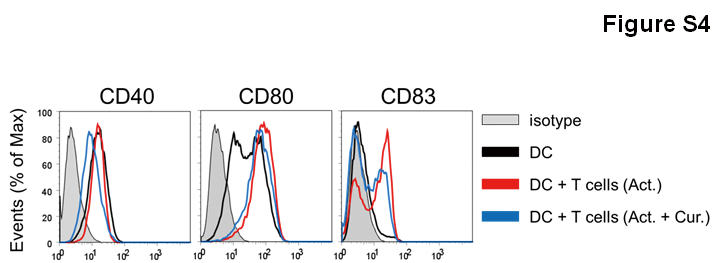

Supplement: Figure S4 — The regulatory effect of CD4+ T cells treated with curcumin and co-cultured with DCs. CD4+ cells were activated with anti-CD2/CD3/CD28 antibody-coated beads (1∶10 for bead-to-cell ratio) with/without 2 µg/mL of curcumin for 5 days with changing fresh media every 3 days, and then co-cultured with DCs for additional 1 day. Autologous DCs were derived from human CD14+ monocyte and treated with IL-4 and GM-CSF for 5 days. Before the co-culture, CD4+ T cells and DCs were washed with PBS. The expression of CD40, CD80 and CD83 on CD11c+ DCs were determined by flow cytometric analysis. (TIF) [file pone.0062300.s004.tif]
